# Unilateral Blepharoptosis Combined With Malignant Exophthalmos After Botulinum Toxin Injection to Glabellar Lines: A Case Report

**DOI:** 10.1111/jocd.70028

**Published:** 2025-02-07

**Authors:** Junli Zou, Zhen Li, Xiaozhen Li, Hong Cai

**Affiliations:** ^1^ Department of Dermatology Air Force Medical University Air Force Medical Center Beijing China

**Keywords:** blepharoptosis, botulinum toxin‐A, complications, malignant exophthalmos, psychological obsessions, thyroid‐associated ophthalmopathy

## Abstract

**Background:**

The ocular adverse reactions caused by the diffusion of BoNT‐A injection are self‐limiting, with symptoms ameliorating over time. Thyroid‐associated ophthalmopathy (TAO) is an autoimmune disease characterized by symptoms such as exophthalmos, diplopia, and eyelid retraction, which may overlap the side effects of BoNT‐A injections. It is crucial to consider other ocular diseases when assessing side effects after BoNT‐A injection, particularly in patients with a history of thyroid disease.

**Aims:**

To describe a complicated case of concurrent malignant exophthalmos and unilateral blepharoptosis following an additional dosage of BoNT‐A injection for glabellar wrinkles.

**Patient:**

A 58‐year‐old female patient received BoNT‐A injection for glabellar wrinkles. Two weeks later, the patient requested an additional dose of BoNT‐A to enhance the effects, receiving an extra 8 units BoNT‐A in the brow muscles. Twenty days later, she presented with a ptosis in the left eye, which progressively manifested into symptoms of exophthalmos, uncoordinated movement, blurred vision, and double vision. Ultimately, she was diagnosed with malignant exophthalmos.

**Results:**

The blepharoptosis following BoNT‐A injection typically manifests a significant improvement within 1–3 months. However, if the symptoms of ptosis persist without evident amelioration and are accompanied by additional ocular discomfort, particularly in patients with thyroid disease, the potential presence of concomitant TAO should be considered.

**Conclusions:**

This report highlights the potential relationship between botulinum toxin and thyroid disease, emphasizing the complexity of complications following cosmetic injections. Clinicians should carefully take the history of a patient, thereby facilitating better identification and management of potential complications.

## 1 Introduction

Botulinum toxin‐A (BoNT‐A) is an injectable neuromodulator that blocks the conduction between the muscle and peripheral nerves by means of inhibiting acetylcholine release that temporarily paralyzes the muscle and reduces the wrinkles [1]. BoNT‐A plays a pivotal role in the cosmetology field since 2002 when the United States Food and Drug Administration (FDA) first approved its use for treating moderate to severe glabellar lines [2]. BoNT‐A injection is a common and effective mainstay of therapy for improving glabellar lines with the advantage of rapid action and fewer side effects. Possible adverse reactions of BoNT‐A at the injection sites include bleeding, bruising, swelling, erythema, and pain, which can gradually reduce within about a week [3]. Occasional systemic effects such as headaches may also occur, but they always improve within 2–4 weeks. The most severe adverse effect is blepharoptosis induced by the local spread of BoNT‐A, which generally disappears spontaneously after 1–3 months [4]. If the side effects of post‐injection mentioned above persist for more than a month without improvement, the possibility of combined other complications should be considered. In this case, we described the process of interaction between the adverse effects of BoNT‐A and new complications to improve our understanding of the diversity and complexity of postoperative adverse reactions, avoid misdiagnosis, and provide evidence for clinical diagnosis.

## 2 Case Report

A 58‐year‐old woman with no past drug allergy or past ocular history visited our hospital on March 22, 2021 for her session of BoNT‐A injections to remove her glabella lines. The patient reported she had a history of thyroid disease and antithyroid therapy but was in stable condition for many years without recurrence. Each vial of 100 U of BoNT‐ A (Botox‐A; American Allergan) was diluted with 2.5 mL of 0.9% normal saline. A total of 7 points were designed in corrugator supercilii, depressor supercilia, and procerus, respectively. Two injection points were designed at the head and abdomen of the corrugator supercilia, respectively, totaling 4 points on both sides. One injection point was designed for the depressor supercilia on each side and an additional point was designed for the procerus specifically. 0.2 mL of solution (8 units) was injected at 4 points of the corrugator supercilia, with each point receiving 0.05 mL (2 units). 0.05 mL of solution (2 units) was injected at 2 points of the depressor supercilia, with each point receiving 0.025 mL (1 unit). 0.05 mL of solution (2 units) was injected into the procerus muscle. Fourteen days later, the patient presented to our hospital for review, expressing dissatisfaction with the post‐injection results. A total of 8 U of additional dose was given in the belly of bilateral corrugator supercilii with an injection volume of 0.05 mL containing 2 U of BoNT‐A (Botox‐A; American Allergan) for designed 4 points totally after informing the relative risk. After 20 days, the patient reported weakness in lifting and limitation in movement of the left eye, while the right eye remained unaffected.

**FIGURE 1:**
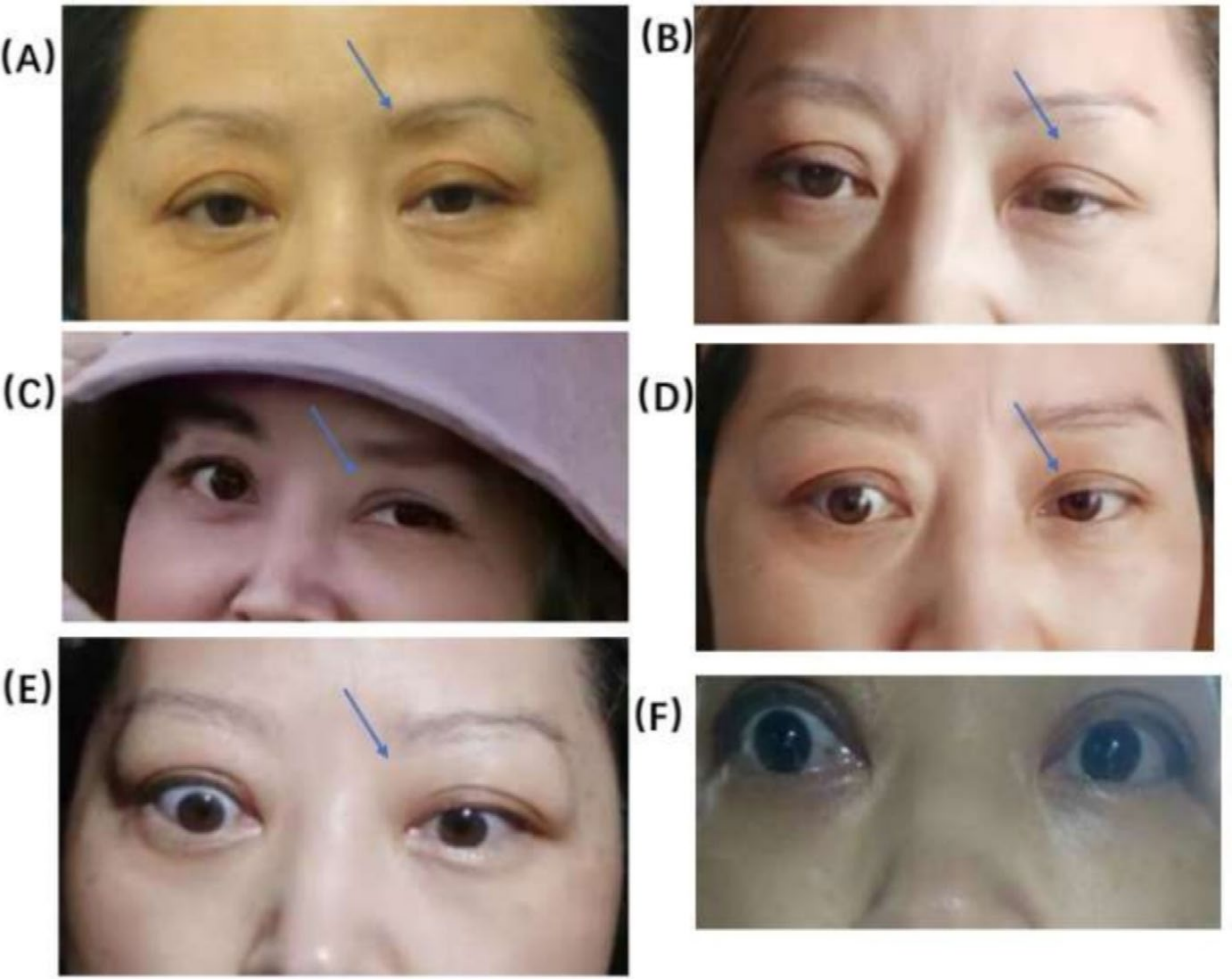
(A) Patient before BoNT‐A injections. (B) 20 days after the additional dose of BoNT‐A was given. (C) More than 1 month after the additional dose of BoNT‐A was given. (D) More than 3 months after the additional dose of BoNT‐A was given. (E‐F) 5 months after the additional dose of BoNT‐A was given.

The preoperative photograph above showed that the patient already exhibited mild ptosis, with the left eye displaying a slightly more prominent drooping compared to the right (Figure 1A). However, the degree of ptosis in the patient's left eye had shown a more obvious aggravation than its pre‐injective state at present (Figure 1B). Give the presentation of the asymmetry in the eyes, along with the noticeable droop in the left eye, it is possible that the BoNT‐A diffuse to the palpebralis muscle, resulting in ptosis OS. Presently, there is no particular therapy for ptosis from BoNT‐A injection. Proposed therapeutic techniques such as eye muscle exercises and Oxymetazoline HCl 0.1% eye drops were suggested to the patient for helping facilitate muscle recovery and reduce the duration of eyelid ptosis. A month later, the patient reported that her condition had been aggravated by the appearance of double vision, un—coordinated movement of the eye, accompanied by obvious eye pain. As evident from the photograph, the difference in the size of the two eyes was noticeable. The left eyelid was still drooping while the right eye appeared more energetic in comparison (Figure 1C). Given the advanced age of the patient and the short time of recovery, it's still deemed that the ptosis was attributed to the diffusion of botulinum toxin. The patient was advised to continue with ocular muscle exercises. After an additional 2 months, the patient reported that her condition had been deteriorating with dizziness and impossible focus when she opened her eyes wide. It is now about 3 months since the initial injection, and the patient's symptoms had become worse rather than eased, which contradicted the conclusion that the effect of BoNT‐A decreases with time. Additionally, the patient's right eye was obviously prominent from the photo and the white of the eye was increased (Figure 1D). Given her history of hyperthyroidism, it is possible that she had experienced a recurrence of hyperthyroidism or an intraocular tumor. The patient was advised to visit the hospital emergency department for ophthalmology consultation. However, she declined the proposal, with the results demonstrating that she did not suffer from hyperthyroidism but from slight hypothyroidism in the setting of the regular thyroid examinations every year, which made the patient even more convinced that her eye problem was directly related to the previous injection treatment.

Starting 5 months after the initial injection, the patient's ocular condition deteriorated further with obvious proptosis, uncoordinated eye movement, blurred vision, and difficulty in focusing. Unable to endure this constant torture any longer, she visited a specialist hospital for a series of tests, and finally, she was diagnosed with malignant exophthalmos.

## 3 Discussion

Botulinum toxin type A (BoNT‐A) injections for glabellar lines is generally considered safe. In the largest multi‐department retrospective study conducted to date, Kim et al. reported that the incidence of adverse events after BoNT‐A injection was only 3.73% [5]. The complications associated with glabellum injections include ptosis, eyebrow asymmetry, diplopia and blurred vision, etc. Among them, blepharoptosis is the most common adverse effect, with the symptoms normally tending to alleviate over time. Using Oxymetazoline HCl 0.1% eye drops and performing eye muscle exercises is helpful in relieving symptoms. In this particular instance, the patient's condition, with a month‐long regimen of ocular muscle exercises, became aggravated instead of improved, prompting the consideration of other concurrent complications. As time elapsed, the patient gradually manifested prominent symptoms of diplopia, accompanied by ocular protrusion. According to these symptoms, the preliminary judgment was that malignant exophthalmos occurred during ptosis.

Malignant exophthalmos is now termed thyroid‐associated ophthalmopathy (TAO). TAO is an organ‐specific autoimmune disorder that is associated with multiple pathogenic factors. The mechanism of action is not completely understood [6]. Although most frequently associated with hyperthyroidism secondary to Graves, about 10% of patients with TAO are euthyroid or hypothyroid. There is currently limited literature supporting whether botulinum toxin has an impact on thyroid function or leads to the recurrence of hyperthyroidism [7, 8].

An in vitro study conducted by Gregoric revealed the existence of molecular similarity between the molecules of type A botulinum toxin and thyroid autoantigens. Antibodies generated following the injection of botulinum toxin can bind to thyroid stimulating hormone receptor (TSH‐R), ultimately leading to an elevation in levels of thyroid stimulating hormone (TSH) in the blood. The function of TSH is to provoke the thyroid gland into synthesizing and secreting thyroid hormones [9]. Theoretically, patients with Graves would have likely triggered an immune response after receiving botulinum toxin injections. However, except for this case, no other reports have been found to discuss the relationship between botulinum toxin and thyroid function. Among the Patients I have known with a history of hyperthyroidism who had been injected with BoNT‐A, no recurrences of hyperthyroidism have been observed in a prolonged period of follow‐up and revisits either. Whether there is a definite influence between the two needs to accumulate more clinical trial data to verify.

Blepharoptosis is classically attributed to the spreading of botulinum toxin through the fascia of the orbital septum to the levator palpebrae superioris (LPS) muscle in the upper eyelid, often due to the application of botulinum toxin outside the safe zone or to marked dispersion of the toxin from excessive manipulation of the region [10]. Blepharoptosis after glabellar injection is frequently associated with injection techniques, dosages, and concentrations, and is also correlated with anatomic variation and loss of skin elasticity in the patient [10]. The patient, in this case, was of an advanced age (58 years old with poor skin elasticity), exhibited mild upper eyelid muscle weakness and asymmetry between the eyes prior to the injection, with a more prominent ptosis in the left eye compared to the right (Figure 1A). These are all risk factors that contribute to blepharoptosis. Given the findings of the patient did not experience ptosis after the initial injection, it is possible that an overly deep (muscle belly) and over‐dose (2 U) was given to the corrugator supercilii muscle above the pupil during the supplementary injection, resulting in the diffusion of toxin to the LPS muscle.

In this case, blepharoptosis and TAO are intertwined and interact secondary to BoNT‐A injection with each other, intensifying the complexity of the course of the disease and the difficulty of judging postoperative complications. BoNT‐A paralyzed the LPS, exerting a resistance effect on the retraction of upper eyelid retraction caused by TAO, thereby rendering the left‐sided ocular symptoms less prominent [11]. To alleviate the drooping symptoms of the left eye, the patient had been advised to engage in ocular muscle exercises. However, both LPS muscles are innervated by a single midline brainstem nucleus that provides equal bilateral central output, which means the unaffected fellow eyelid would have likely received equal strengthening through these exercises, aggravating the symptoms of the contralateral eyelid and resulting in more pronounced upper eyelid retraction and corneal exposure [12]. The retraction was alleviated in the left upper eyelid by the action of BoNT‐A, while the retraction was intensified in the right upper eyelid by the constant exercise, which made the contrast between the size of the two eyes more apparent. Since the patient's early signs of exophthalmos were not significant, the contrast might easily be misinterpreted as persistent ptosis of the left eyelid. Furthermore, the result of the follow‐up examination showed euthyroid or even a slight hypothyroid also became a reason for the patient to deny the association between eyelid symptoms and thyroid conditions.

## 4 Conclusion

BoNT‐A injection is a routine medical cosmetic technique with low risk and high prevalence. The adverse reactions of BoNT‐A injection are self‐limiting and typically resolve within a week to a month; some may persist for up to 3 months or longer in rare cases. Any other complications should be considered if the patient's symptoms worsen 1 month after receiving BonT‐A injection therapy. When experiencing worsening ocular symptoms over time during BonT‐A injections, the possibility of concurrent TAO should be considered. Although there is currently no conclusive evidence that BonT‐A injections directly induce hyperthyroidism, further clinical observation and data accumulation are still needed to verify whether BonT‐A may accelerate the development of hyperthyroidism and then cause more serious complications.

A prospective diagnosis should be suggested to injectors in the setting of adverse reactions occurring after injections, to avoid the patient amplifying inner anxiety from psychological fear, blaming all discomfort on the failure of the injection rather than the possibility of other combined diseases. It is also crucial to provide patients with patient education, helping them gain a clear understanding of their illness, rectifying their psychological obsession, and motivating them to actively cooperate in undergoing relevant medical examinations to ensure timely treatment offered. Certainly, this also puts forward higher requirements for cosmetic surgeons, who should possess a comprehensive clinical knowledge to make an accurate judgment on the complicated postoperative complications.

## Author Contributions

Junli Zou contributed to drafting the article. Zhen Li revised it critically for important intellectual content. Xiaozhen Li participated in the conception of the article. Hong Cai is the guarantor for the article and oversaw the decision to publish.

## Consent

The patient consented to the publication of the case in writing and orally.

## Conflicts of Interest

The authors declare no conflicts of interest.

## Data Availability

Data sharing not applicable to this article as no datasets were generated or analyzed during the current study.

## References

[1] J. Frevert , “Pharmaceutical, Biological, and Clinical Properties of Botulinum Neurotoxin Type A Products,” Drugs in R&D 15, no. 1 (2015): 1–9, 10.1007/s40268-014-0077-1. 25559581 PMC4359186

[2] R. Small , “Botulinum Toxin Injection for Facial Wrinkles,” American Family Physician 90, no. 3 (2014): 168–175. 25077722

[3] D. Zargaran , F. Zoller , A. Zargaran , et al., “Complications of Cosmetic Botulinum Toxin A Injections to the Upper Face: A Systematic Review and Meta‐Analysis,” Aesthetic Surgery Journal 42, no. 5 (2022): NP327–NP336, 10.1093/asj/sjac036. 35178552 PMC9005453

[4] B. K. Satriyasa , “Botulinum Toxin (Botox) A for Reducing the Appearance of Facial Wrinkles: A Literature Review of Clinical Use and Pharmacological Aspect,” Clinical and Cosmetic Investigative Dermatology 12 (2019): 223–228, 10.2147/CCID.S202919. PMC6489637 31114283

[5] B. W. Kim , G. H. Park , W. J. Yun , et al., “Adverse Events Associated With Botulinum Toxin Injection: A Multidepartment, Retrospective Study of 5310 Treatments Administered to 1819 Patients,” Journal of Dermatological Treatment 25, no. 4 (2014): 331–336, 10.3109/09546634.2013.789473. 23537074

[6] C. Ma , H. Li , S. Lu , and X. Li , “Thyroid‐Associated Ophthalmopathy and Ferroptosis: A Review of Pathological Mechanisms and Therapeutic Strategies,” Frontiers in Immunology 15 (2024): 1475923, 10.3389/fimmu.2024.1475923. 39712031 PMC11659143

[7] D. Y. Konkova and V. N. Karnaukh , “Sochetanie Miastenii i Autoimmunnoi oftal'mopatii s Yavleniyami Zlokachestvennogo ekzoftal'ma [A Case of the Combination of Myasthenia Gravis and Autoimmune Eye Disease With the Symptoms of Malignant Exophthalmos],” Zhurnal Nevropatologii i Psikhiatrii Imeni S.S. Korsakova 116, no. 10 (2016): 82–84, 10.17116/jnevro201611610182-84. 27845322

[8] T. D. Hoang , D. J. Stocker , E. L. Chou , and H. B. Burch , “2022 Update on Clinical Management of Graves Disease and Thyroid eye Disease,” Endocrinology and Metabolism Clinics of North America 51, no. 2 (2022): 287–304, 10.1016/j.ecl.2021.12.004. 35662442 PMC9174594

[9] E. Gregoric , J. A. Gregoric , F. Guarneri , and S. Benvenga , “Injections of *Clostridium botulinum* Neurotoxin A May Cause Thyroid Complications in Predisposed Persons Based on Molecular Mimicry With Thyroid Autoantigens,” Endocrine 39, no. 1 (2011): 41–47, 10.1007/s12020-010-9410-9. 21061092

[10] M. S. Nestor , H. Han , A. Gade , D. Fischer , Y. Saban , and R. Polselli , “Botulinum Toxin‐Induced Blepharoptosis: Anatomy, Etiology, Prevention, and Therapeutic Options,” Journal of Cosmetic Dermatology 20, no. 10 (2021): 3133–3146, 10.1111/jocd.14361. 34378298 PMC9290925

[11] Y. Xie , Q. Liu , L. Li , et al., “DaxibotulinumtoxinA for Injection to Treat Moderate or Severe Glabellar Lines: A Randomized, Multicenter, Phase III, Double‐Blind, Placebo‐Controlled Trial in China,” Journal of Plastic, Reconstructive & Aesthetic Surgery 99 (2024): 67–75, 10.1016/j.bjps.2024.09.012. 39353286

[12] A. D. Chen , Y. W. Lai , H. T. Lai , et al., “The Impact of Hering's Law in Blepharoptosis: Literature Review,” Annals of Plastic Surgery 76, no. 1 (2016): S96–S100, 10.1097/SAP.0000000000000689. 26808763

